# Supplementary material for: Alphaviral Capsid Proteins Inhibit Stress Granule Assembly via Competitive RNA Binding With G3BP1
Source: Adv Sci (Weinh). 2026 Jan 27;13(20):e17009. doi: 10.1002/advs.202517009 (PMC13067816; doi:10.1002/advs.202517009)
Supplement: Supplementary file 1 — Supporting File 1: advs74010‐sup‐0001‐SuppMat.docx. [file ADVS-13-e17009-s001.docx]

**Figure S1.** Determination of the threshold concentration of SFV Capsid in SG inhibition. (A) Immunofluorescent analysis of SA-induced SG formation in U2OS cells expressing the indicated EGFP-tagged cysteine mutants of SFV capsid-40-110aa. Scale bar, 10 μm. (B) Quantification of the relative fluorescent intensity of SFV capsid in SG inhibition. Data are plotted as a minimum to maximum. n=10 values in the determined thresholding bin. (C) The threshold concentration is shown. See Methods for defining the bin. (D) The representative image of SG disassembly in SFV capsid high-expressed cells, and colocalization of capsid with SG in low-expressed cells. Scale bar, 10 μm. (E) The standard curve of fluorescent intensity and transformation into the absolute SG inhibitory concentration of GFP-SFV capsid. The estimated SG-inhibiting threshold concentration of SFV Capsid is labeled above the curve. (F) Intracellular concentration estimation of total SFV capsid protein at 8, 16, and 24 hpi in SFV-infected U2OS cells (MOI=0.1). The estimated SFV capsid concentration is listed below the curve. (G) Quantification of threshold fluorescence of SFV Capsid-40-110aa lysine valency mutants in SG inhibition. Data are plotted as a minimum to maximum. n=10 values in the determined thresholding bin. Below are the representative images of the indicated mutants in 0.5 mM SA-treated U2OS cells. Scale bar, 10 μm. (H) The standard curve of fluorescent intensity and transformation into the absolute SG inhibitory concentration of GFP-SFV capsid-22-110aa-KR (left). The estimated SG-inhibiting threshold concentration of SFV Capsid is labeled above the curve. Quantification of the relative threshold fluorescence of SFV capsid-22-110aa-KR in SG inhibition (right). Data are plotted as a minimum to maximum. n=10 values in the determined thresholding bin.

**Figure S2.** SFV capsid inhibited SG not by altering SG network. (A, B) Volcano plot of TurboID-G3BP1 proteome with or without SFV capsid in untreated (A) and SA-treated (B) HEK293T cells. (C, D) Heatmap of representative SG protein abundance with or without SFV capsid in untreated (C) and SA-treated (D) HEK293T cells.

**Figure S3.** SFV capsid interacts with various host RBPs and mRNA. (A) Volcano plot of the binding transcripts of capsid in cells challenged with SFV and arsenite by capsid RIP-seq. (B) Venn diagram of SFV N-binding transcripts in cells challenged with SFV and arsenite. (C) Pie charts of capsid binding elements in the context of arsenite treatment (left) or SFV infection (right). (D, E) Venn plots of the SG-enriched transcriptome and the capsid binding transcriptome in SA-treated (D) or SFV-infected cells (E). (F) Venn plots of the SARS-CoV-2_N transcriptome and the SFV-infected capsid binding transcriptome.

**Figure S4.** Amino acid sequence alignment of alphavirus capsids. (A) Alignment of capsid proteins of the alphavirus family members. (B) Alignment of helix-forming region and poly-lysine tract of alphaviral capsid proteins. (C) Phylogenetic tree of the alphavirus family based on the capsid protein sequences.

**Figure S5.** All alphavirus capsids showed SG inhibition. (A) The representative images of SA-induced SG formation in U2OS cells expressing the other 31 alphavirus capsid α-helix poly-lysine fragments. Scale bar, 10 μm. (B) The representative image of SG disassembly in VEEV capsid-expressing cells (top). Quantification of the relative threshold fluorescence of VEEV capsid in SG inhibition. Data are plotted as a minimum to maximum. n=10 values in the determined thresholding bin (bottom, left). The standard curve of fluorescent intensity and transformation into the absolute SG inhibitory concentration of GFP-VEEV capsid (bottom, right). The estimated SG-inhibiting threshold concentration of VEEV Capsid is labeled above the curve. Scale bar, 10 μm. (C) Representative immunofluorescent images of VEEV capsids with different tags; the no-tagged VEEV capsid was stained with an SFV capsid antibody. Scale bar, 10 μm. (D) Confocal imaging of GFP-VEEV infected U2OS cells co-stained with NPM1 antibody. Two representative patterns were shown. Scale bar, 10 μm. (E) Confocal imaging of GFP-VEEV infected HA-VEEV capsid expressing U2OS cells. Scale bar, 10 μm.

**Figure S6.** Many other viral nucleocapsids do not share the feature in SG inhibition. (A) Immunofluorescent analysis of SA-induced SG formation in U2OS cells expressing the mCherry-tagged VSV_N protein. Scale bar, 10 μm. (B) Quantification of SG area and number related to (A). Data are plotted as minimum to maximum; lines indicate the first quartile (lower), median, and the third quartile (upper) with n≥50 for each sample (Student’s t-test, unpaired, two-tailed). ***p < 0.001. Data are from one representative experiment for at least two independent experiments. (C) The *in vitro* LLPS of 50 μM G3BP1 and 100 ng/μl polyA RNA in the presence of increasing concentrations of VSV_N. Scale bar, 10 μm. (D) Immunofluorescent analysis of SA-induced SG formation in U2OS cells expressing the EGFP-tagged DENV2, WNV, and ZIKV capsid proteins. Scale bar, 10 μm. (E) Quantification of SG area and number related to (D). Data are plotted as minimum to maximum; lines indicate the first quartile (lower), median, and the third quartile (upper) with n≥50 for each sample (one-way ANOVA with Mixed-effects analysis). ****p < 0.0001, ns is not significant. Data are from one representative experiment for at least two independent experiments. (F) The structure alignment of SFV capsid N-terminus with an online Foldseek tool (<https://search.foldseek.com/search>)^56^. The viral proteins sharing structural similarities with SFV are highlighted in red.

**Figure S7.** HIV-1 nucleocapsid inhibits SG formation. (A) Schematic illustration of HIV-1 gag protein domains, with PONDR analysis and Alphafold prediction of HIV-1 nucleocapsid. (B) Schematic illustration of the indicated HIV-1 nucleocapsid lysine to alanine mutants and ZF-disruptive mutants. (C) Immunofluorescent analysis of SA-induced SG formation in U2OS cells expressing the indicated EGFP-tagged HIV-1 gag, capsid, nucleocapsid protein WT, KA, and ZF mutants. Scale bar, 10 μm. (D) Quantification of SG area and number of SG related to (C). Data are plotted as minimum to maximum; lines indicate the first quartile (lower), median, and the third quartile (upper) with n≥50 for each sample (one-way ANOVA with Mixed-effects analysis). ****p < 0.0001, ns is not significant. Data are from one representative experiment for two independent experiments.

**Figure S8.** Proteome-wide search for capsid-like candidates. (A) A list showing 30 identified hits that contain an IDR with a lysine ratio over 30%. Highlighted genes are experimentally tested. (B) Representative confocal images of SA-induced SG formation in U2OS cells expressing the proteins with highly enriched lysine residues as listed in (A).

**Figure S9.** SFV capsid peptide inhibits stress granule formation induced by ALS-associated mutation. (A) Immunofluorescent analysis of FUS ΔPY-induced spontaneous SG formation in U2OS cells expressing the indicated mCherry-tagged SFV Capsid-40-110aa, 66-110aa, 40-110aa-2LP. (B) Quantification of cells with spontaneous SG in the indicated mCherry fusion-positive cells. Data are mean ± SEM with n=3 replicates. ****p < 0.0001, ns is not significant. Data are from one representative experiment for three independent experiments.
