# Supplementary figures and images for: Alphaviral Capsid Proteins Inhibit Stress Granule Assembly via Competitive RNA Binding With G3BP1
Source: Adv Sci (Weinh). 2026 Jan 27;13(20):e17009. doi: 10.1002/advs.202517009 (PMC13067816; doi:10.1002/advs.202517009)

**Figure S1. Determination of the threshold concentration of SFV CP in SG inhibition**

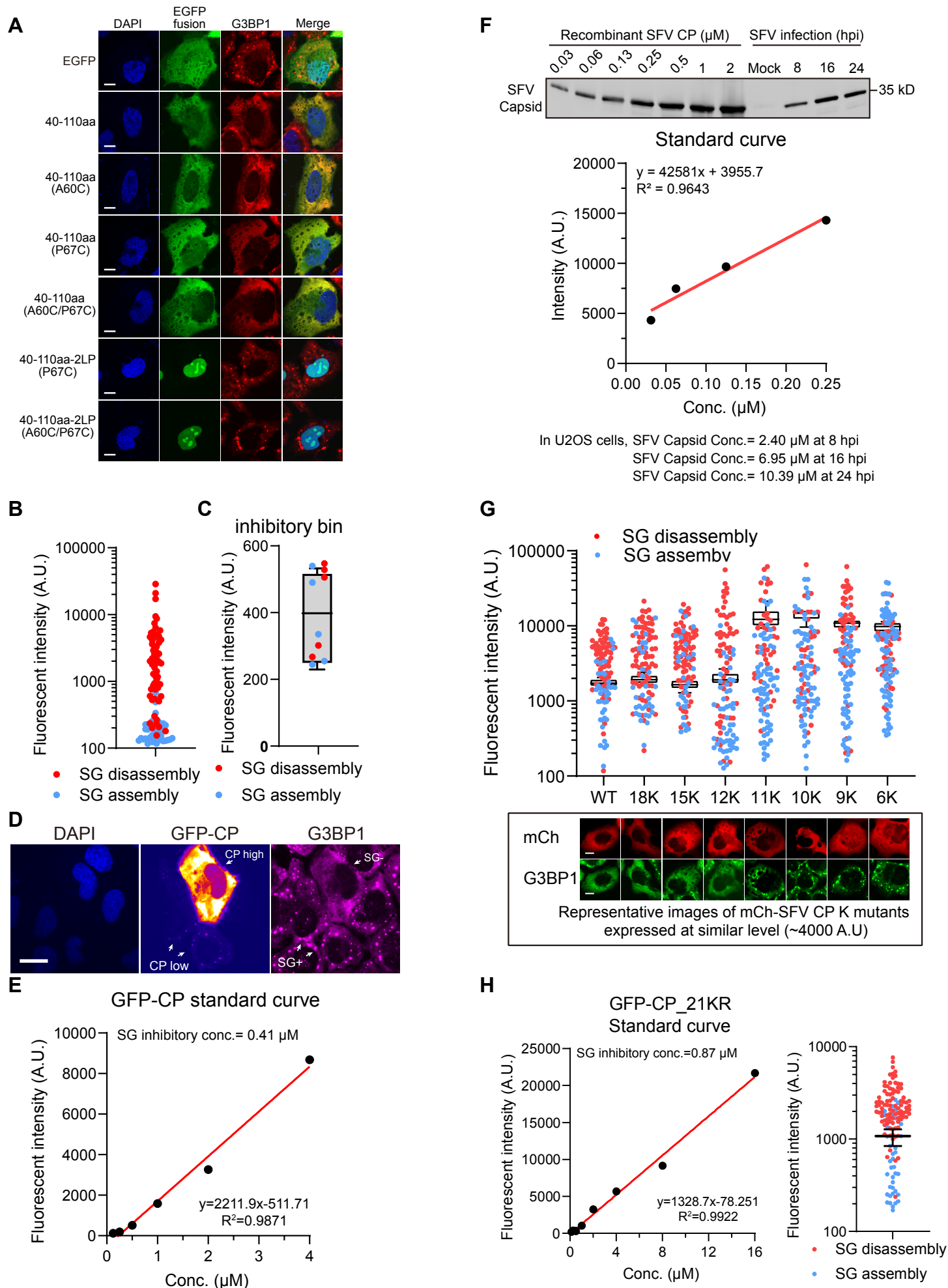

Supplement: Supplementary file 2 — Supporting File 2: advs74010‐sup‐0002‐FigureS1.pdf. [file ADVS-13-e17009-s004.pdf]

### Figure S2. SG inhibition by SFV Capsid is not through altering SG network

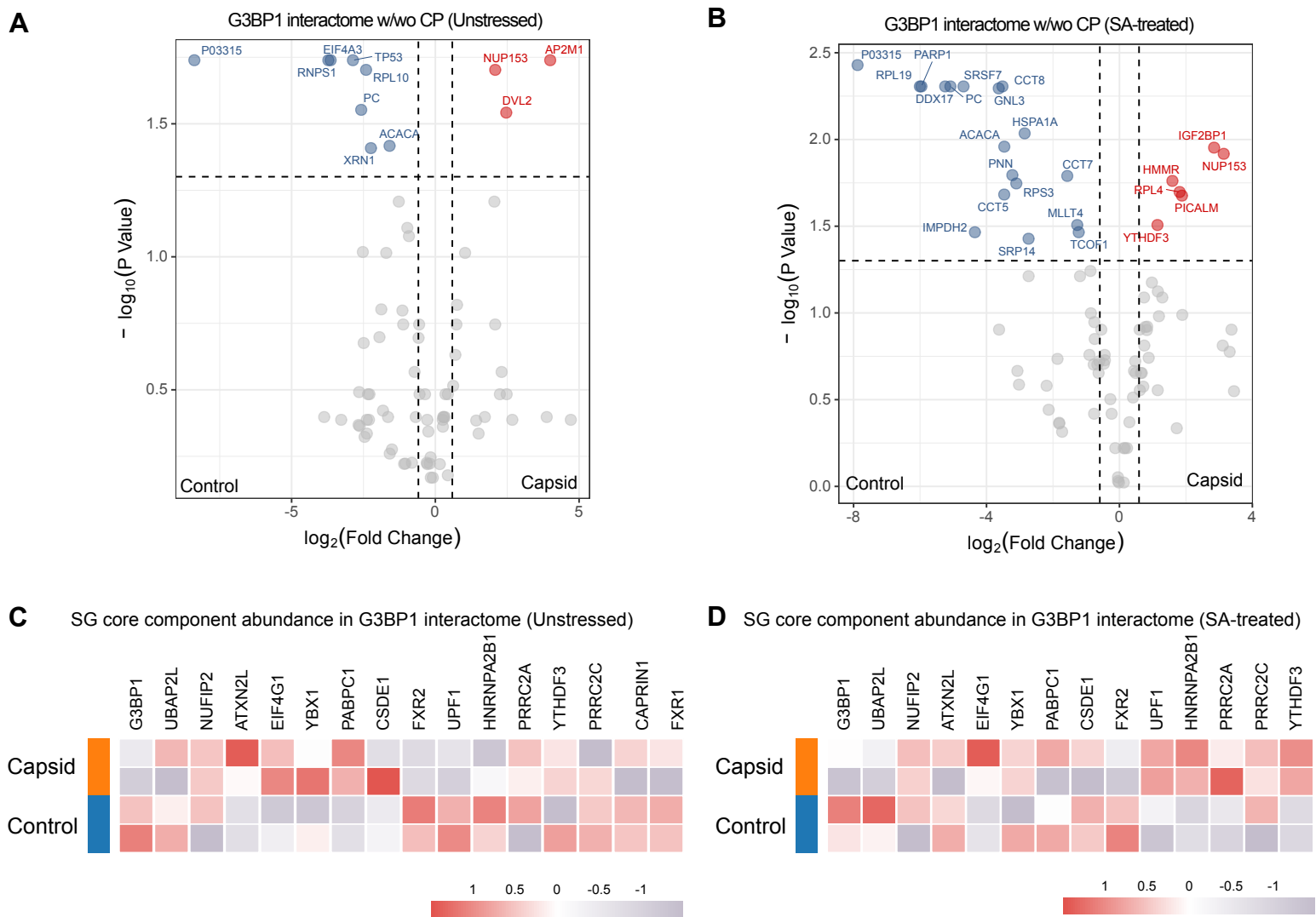

Supplement: Supplementary file 3 — Supporting File 3: advs74010‐sup‐0003‐FigureS2.pdf. [file ADVS-13-e17009-s002.pdf]

Figure S3. SFV CP interacts with various host RBPs and mRNA

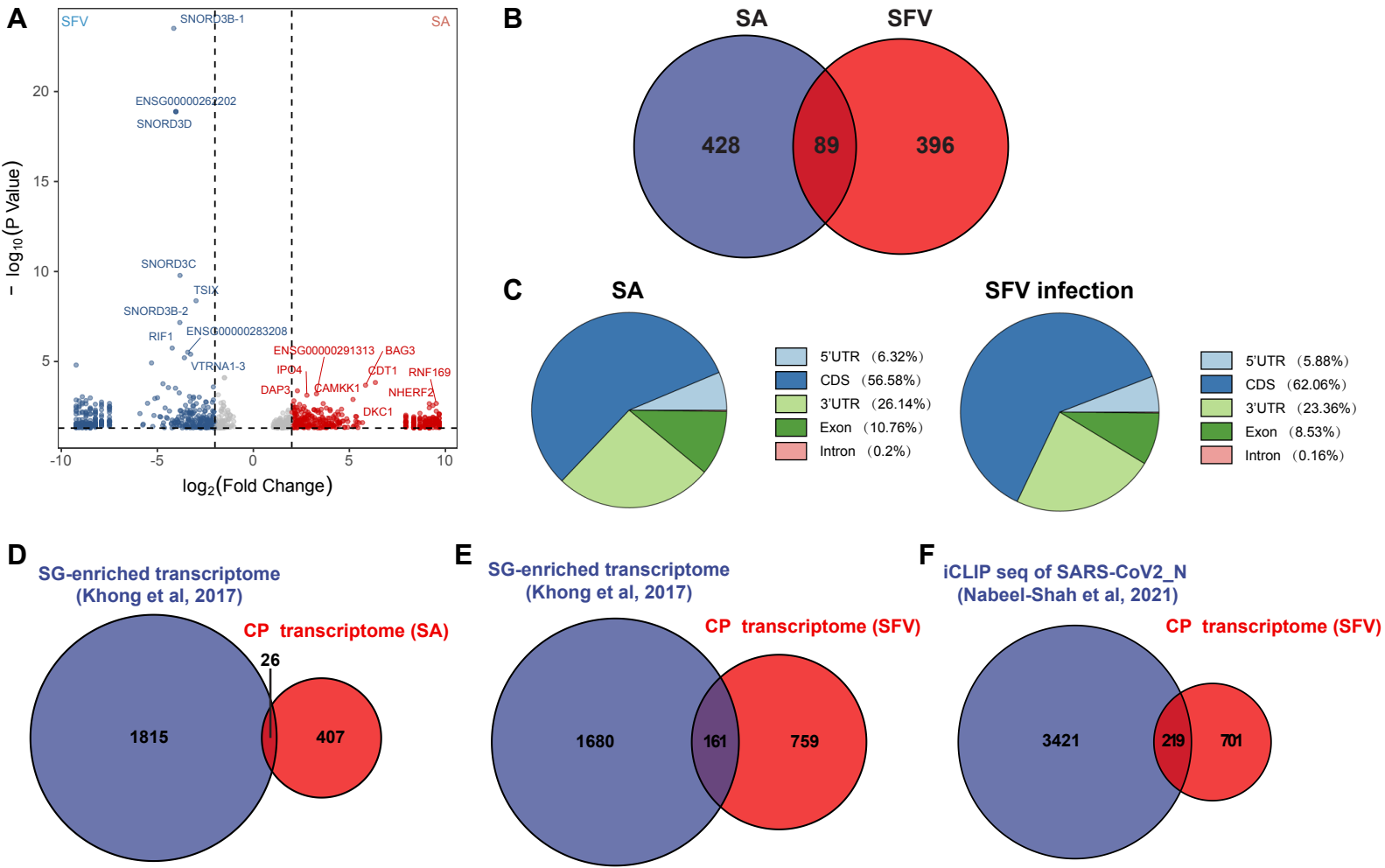

Supplement: Supplementary file 4 — Supporting File 4: advs74010‐sup‐0004‐FigureS3.pdf. [file ADVS-13-e17009-s010.pdf]

Figure. S4 Amino acid sequence alignment of alphavirus capsids

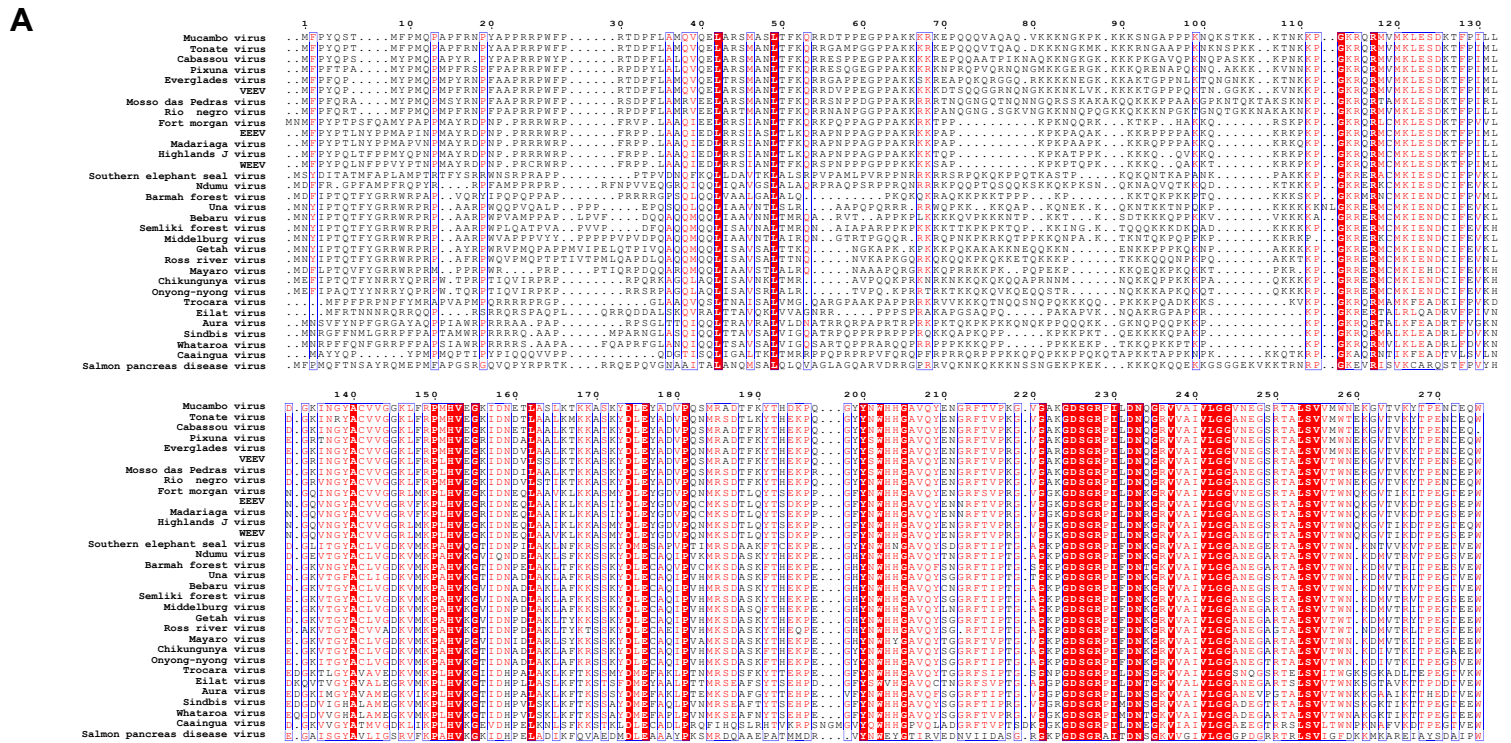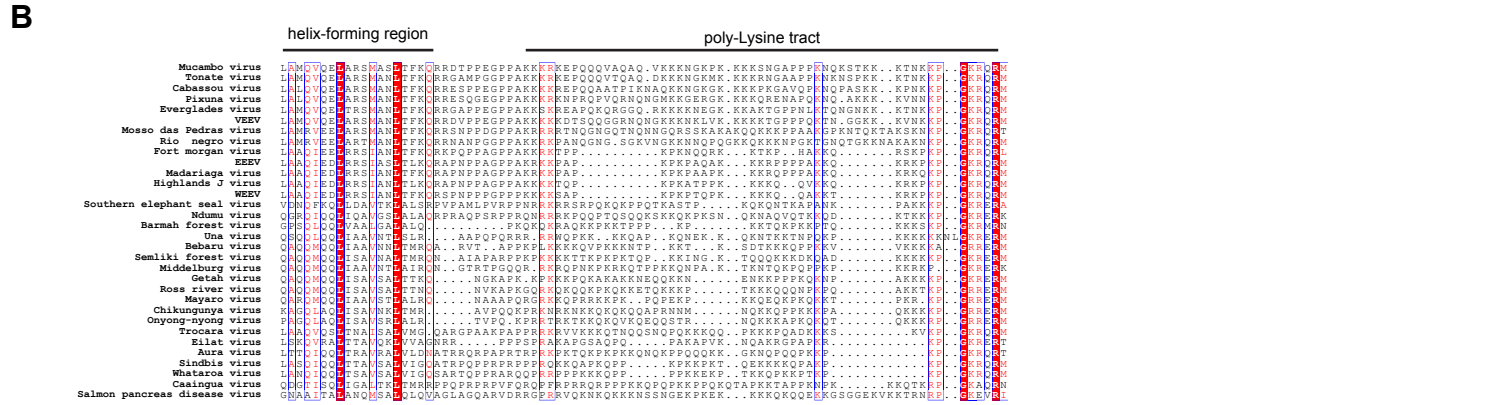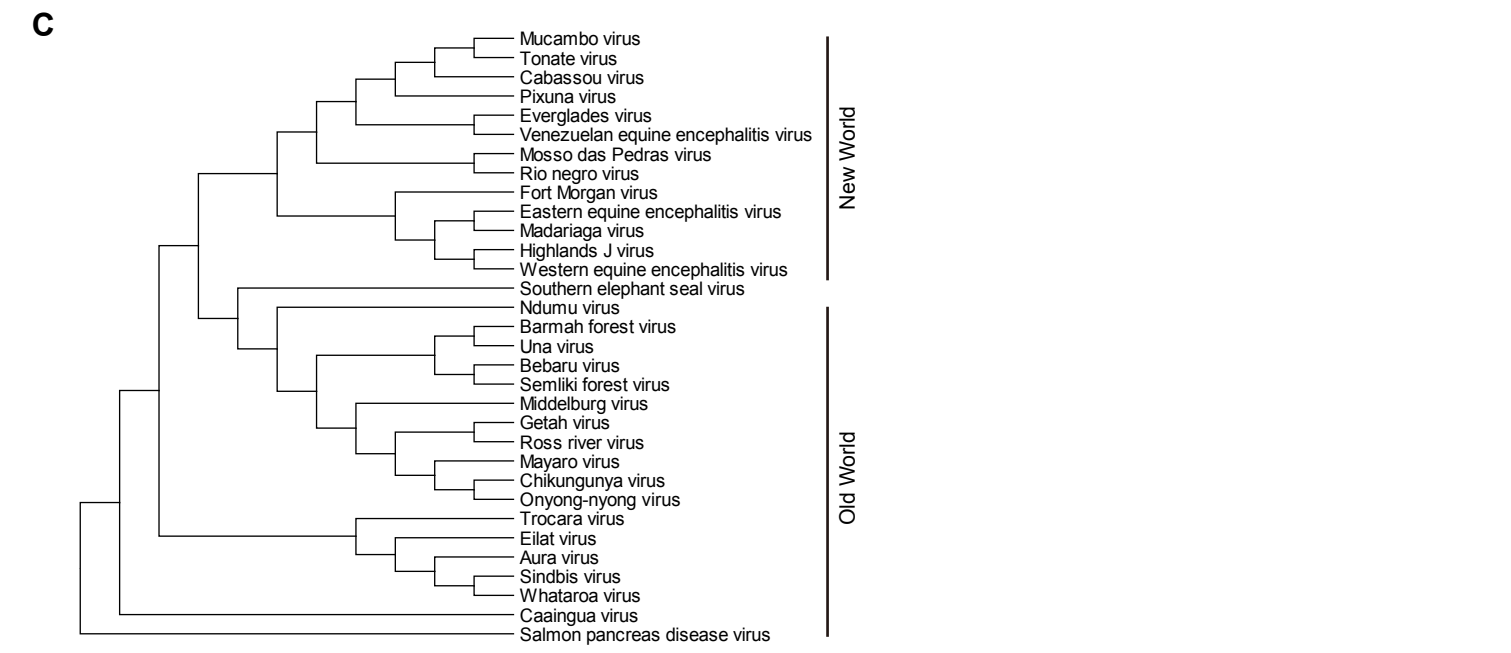

Supplement: Supplementary file 5 — Supporting File 5: advs74010‐sup‐0005‐FigureS4.pdf. [file ADVS-13-e17009-s009.pdf]

Figure S5. All alphavirus capsid fragments showed SG inhibition in cells

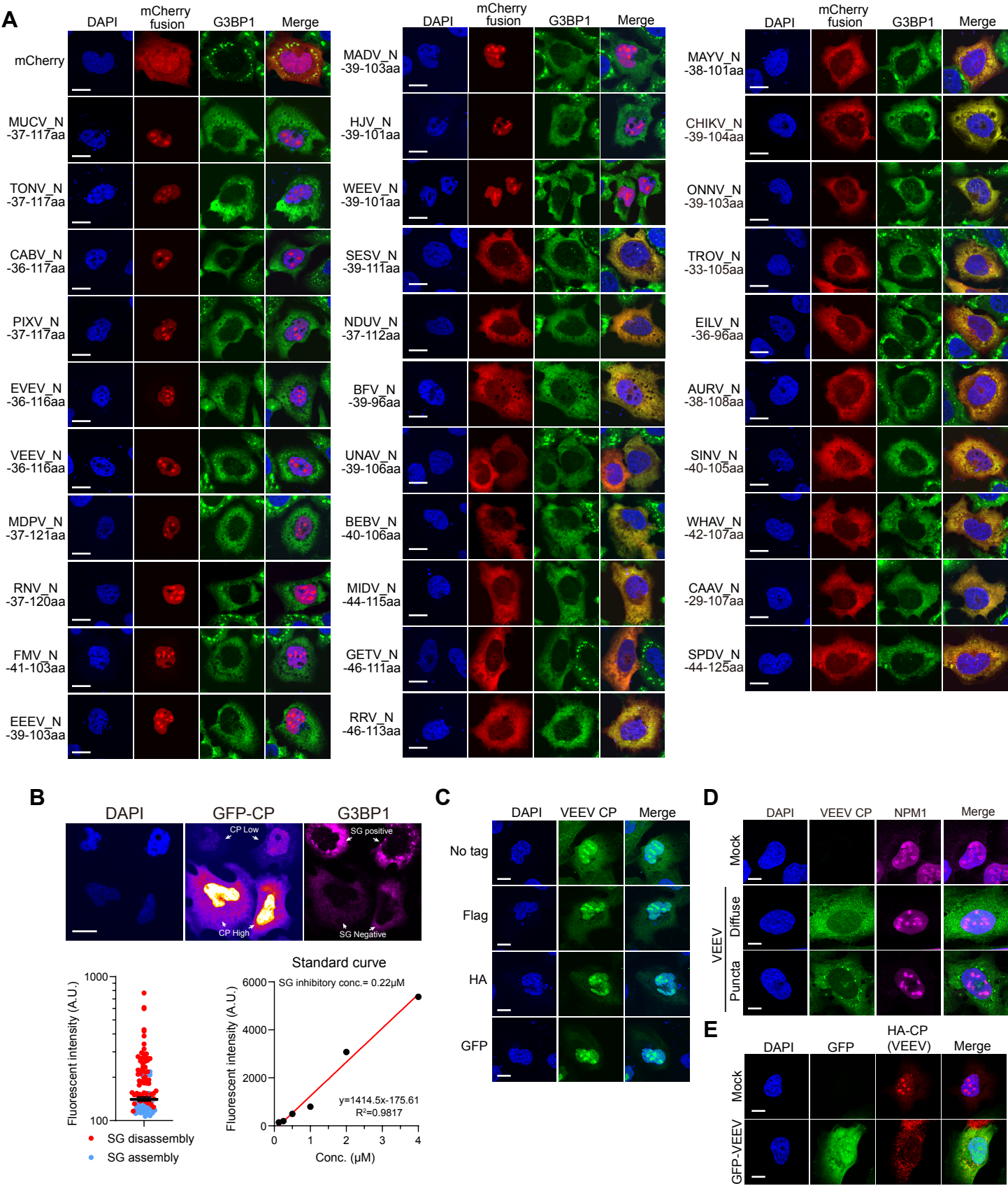

Supplement: Supplementary file 6 — Supporting File 6: advs74010‐sup‐0006‐FigureS5.pdf. [file ADVS-13-e17009-s003.pdf]

Figure S6. Many other viral nucleocapsids do not share the feature in SG inhibition

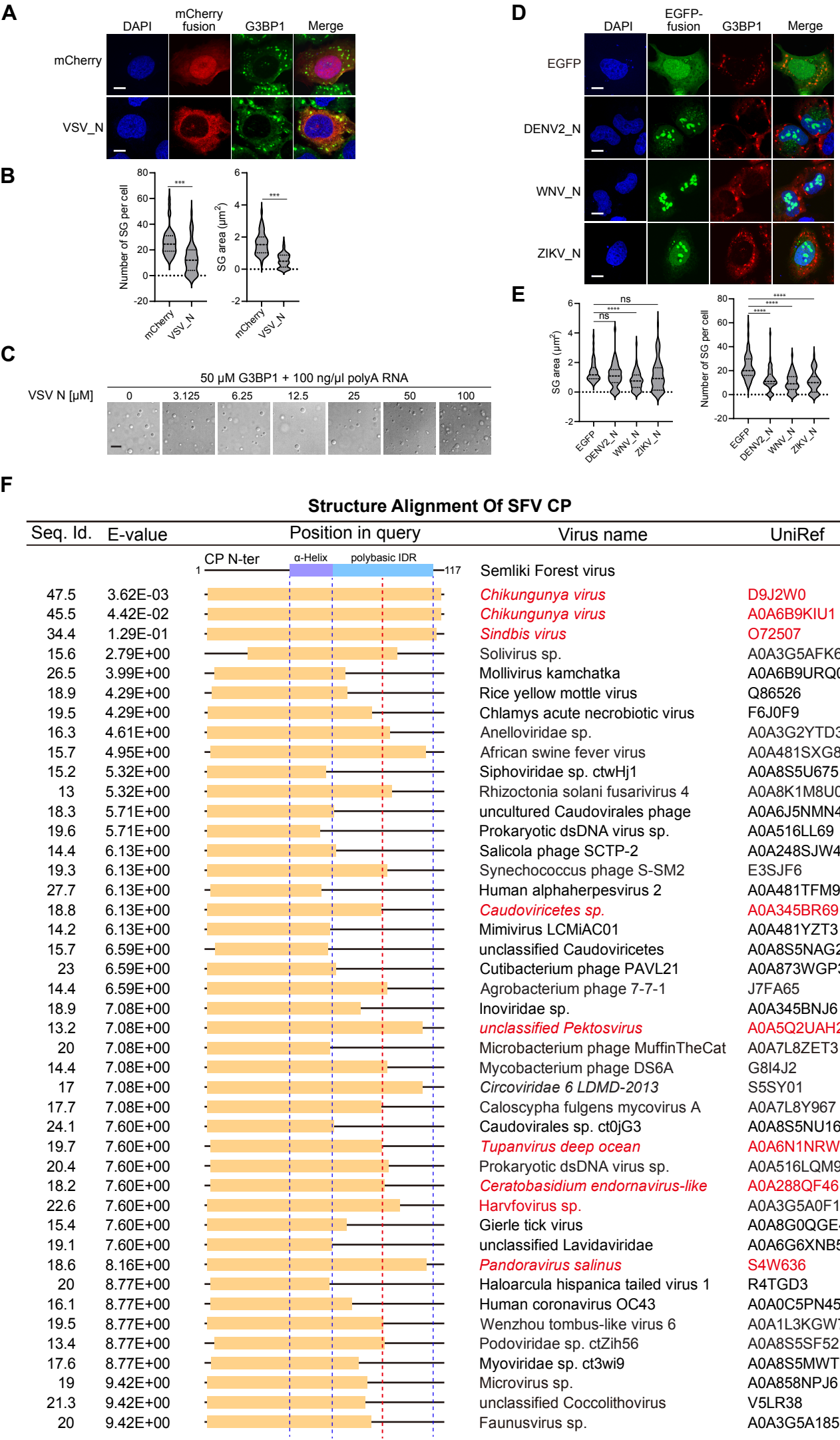

Supplement: Supplementary file 7 — Supporting File 7: advs74010‐sup‐0007‐FigureS6.pdf. [file ADVS-13-e17009-s006.pdf]

Figure S7. HIV-1 nucleocapsid inhibits SG formation

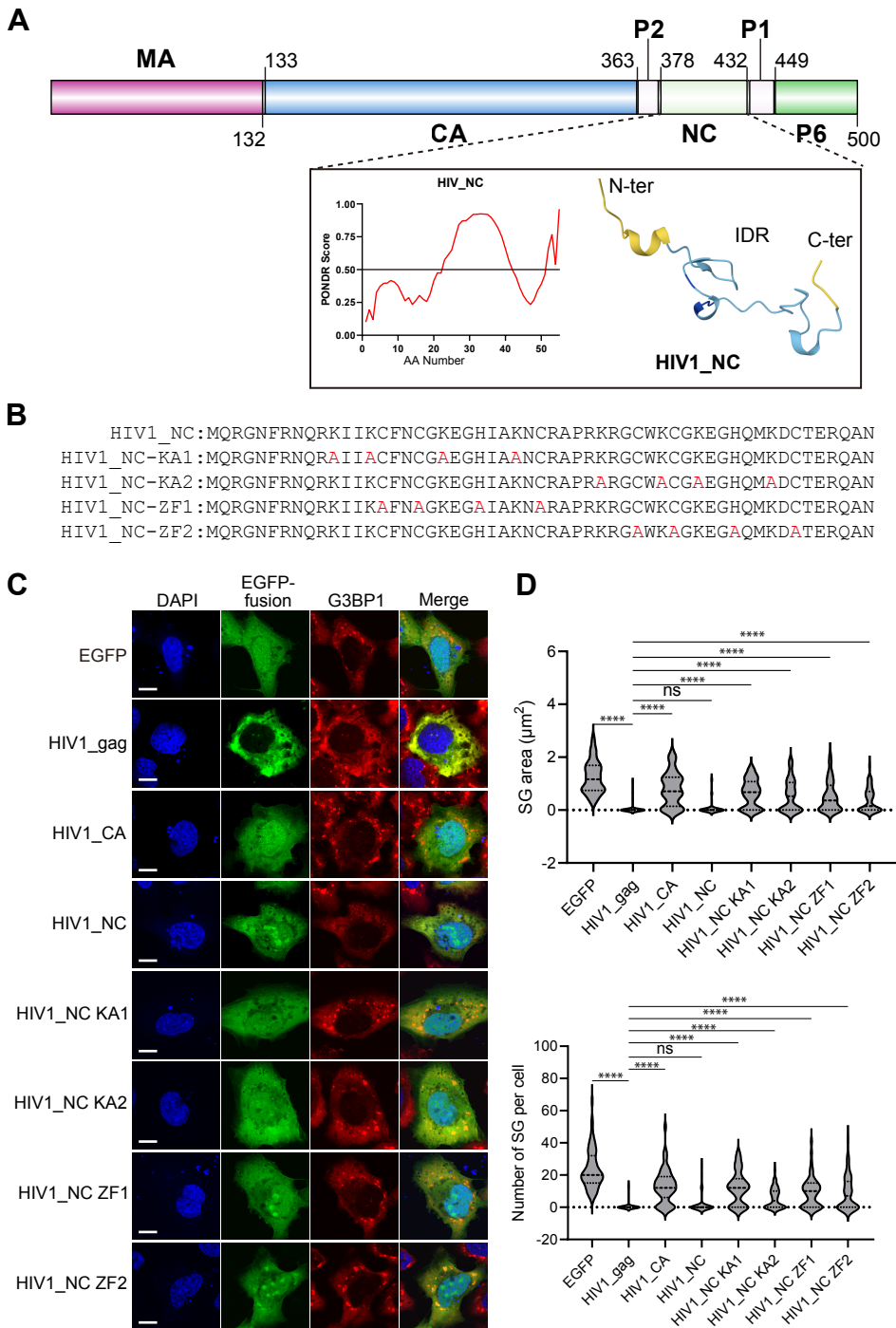

Supplement: Supplementary file 8 — Supporting File 8: advs74010‐sup‐0008‐FigureS7.pdf. [file ADVS-13-e17009-s005.pdf]

**Figure S9. SFV capsid peptide inhibits SG induced by ALS-associated mutation**

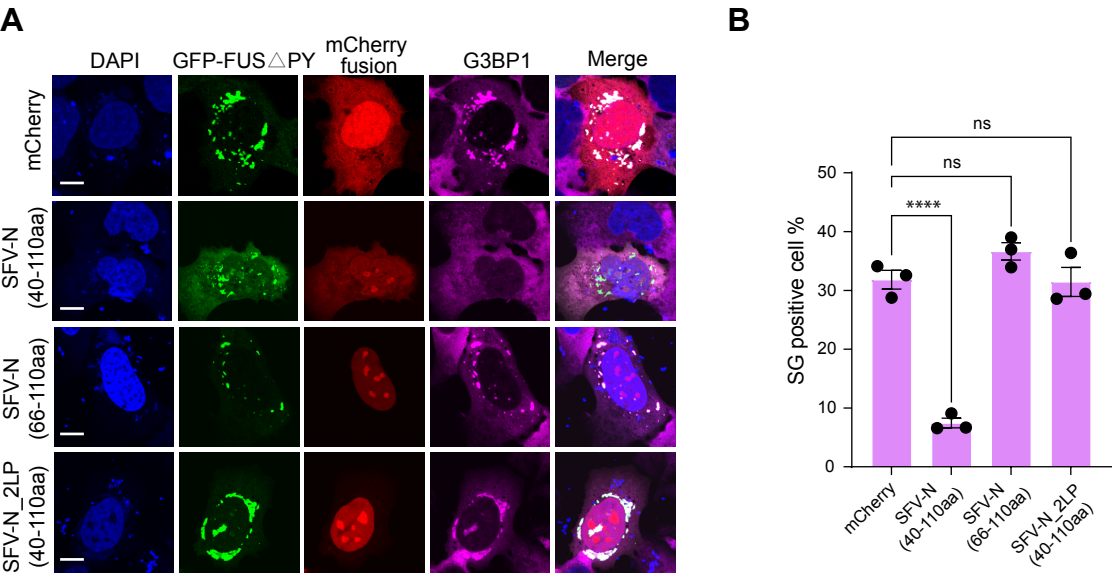

Supplement: Supplementary file 10 — Supporting File 10: advs74010‐sup‐0010‐FigureS9.pdf. [file ADVS-13-e17009-s007.pdf]

Supplemental Figure 10. Uncropped images for WB in this study

Fig. 3K

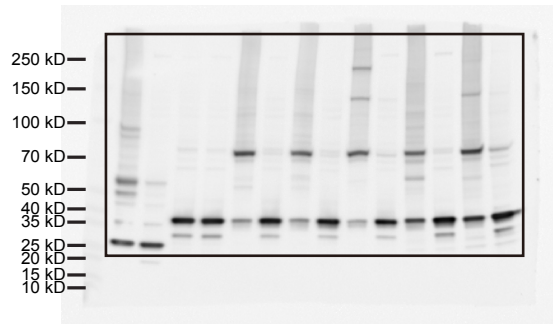

Fig. 6H

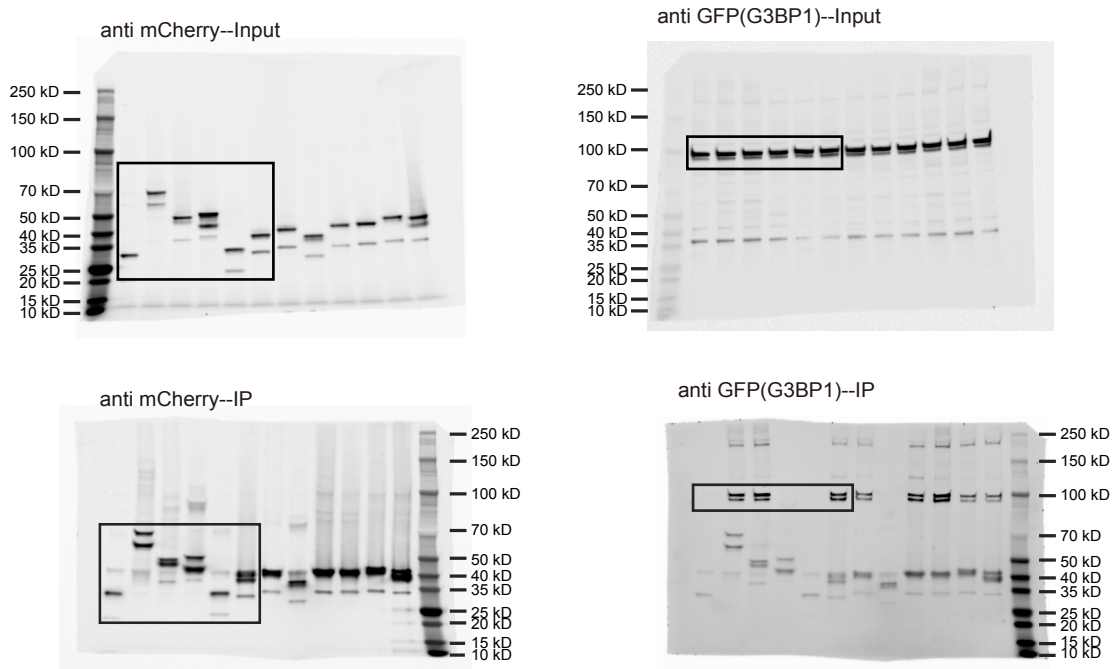

Fig. 6I

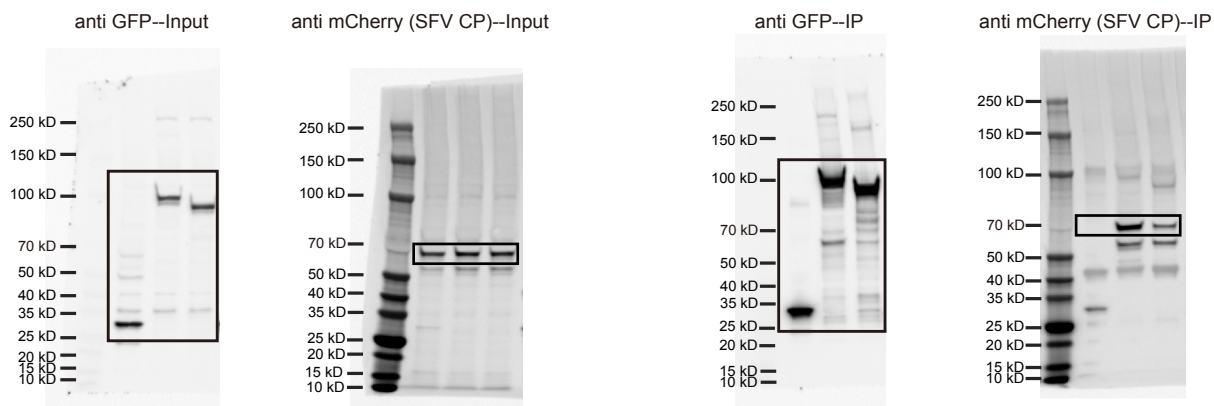

Fig. 7I

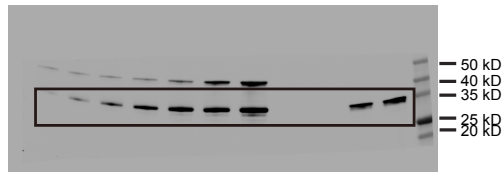

Fig. S1F

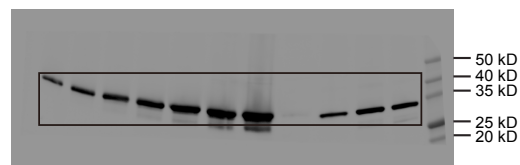

Supplement: Supplementary file 11 — Supporting File 11: advs74010‐sup‐0011‐FigureS10.pdf. [file ADVS-13-e17009-s011.pdf]
