# Supplementary material for: Alphaviral Capsid Proteins Inhibit Stress Granule Assembly via Competitive RNA Binding With G3BP1
Source: Adv Sci (Weinh). 2026 Jan 27;13(20):e17009. doi: 10.1002/advs.202517009 (PMC13067816; doi:10.1002/advs.202517009)

Figure S8. Human proteins enriched with lysine don't show SG inhibition

A

Human proteome-wide identification of IDRs enriched of lysines (min 30%)

Protein name	Uniprot accession	Lys Ratio (%)	IDR Sequence
LRRIQ4	A6NIV6	40.00	QRGFGKFGELLKPQKKGKTSPKDKKGKKDVKGKPGKGKKK
SMARCA5	O60264	34.29	KEKAEKKKRGPKPSTQKRKMDGAPDGRGRKKKLKL
H2BC11	P06899	35.48	MPEPAKSAPAPKKGSKKAVTKAQKKDGKKRK
H1-0	P07305	42.71	DEPKKSVAFKKTKKEIKKVATPKKASKPKKAASKAPTKKPKATPVKKAKKKLAATPKKAKKPKTVKAKPVKASKPKKAKPVKPKAKSSAKRAGKKK
HSP90AA1	P07900	31.37	EAEEKEDKEEEKEKEEKESEDKPEIEDVGSDEEEEKKDGDKKKKKKIKEKY
PARP1	P09874	32.43	KKQLPGVKSEGKRKGDEVDGVDEVAKKKSKKEKDKDS
H1-4	P10412	39.81	ASGEAKPKAKKAGAAKAKKPAGAAKKPKKATGAATPKKSAKKTPKKAKKPAAAAGAKKAKSPKKAKAAKPKKAPKSPAKAKAVKPKAAKPKTAKPKAAKPKKAAAKKK
H1-5	P16401	39.82	AASGEAKPKAKKAGAAKAKKPAGATPKKAKKAAGAKKAVKKTPKKAKKPAAAGVKKVAKSPKKAKAAAKPKKATKSPAKPKAVKPKAAKPKAAKPKAAKPKAAKAKKAAAKKK
H1-3	P16402	37.27	AASGEGKPKAKKAGAAKPRKPAGAAKKPKKVAGAATPKKSIKKTPKKVKKPATAAGTKKVAKSAKKVKTPQPKKAAKSPAKAKAPKPKAAKPKSGKPKVTKAKKAAPKKK
H1-2	P16403	39.22	ASGEAKPKVKKAGGTKPKKPVGAAKKPKKAAGGATPKKSAKKTPKKAKKPAAATVTKKVAKSPKKAKVAKPKKAAKSAAKAVKPKAAKPKVVKPKKAAPKKK
PTN	P21246	38.71	SKPCGKLTKPKPQAESKKKKKEGKKQEKMLD
RPL4	P36578	32.35	ALQAKSDEKAAVAGKKPVVGKKGKKAAVGVKKQKKPLVGKKAAATKKPAPEKKPAEKKPTTEEKKPAA
RPS25	P62851	42.11	MPPKDDKKKKDAGKSAKKDKDPVNKSGGKAKKKKWSKG
H1-1	Q02539	36.89	KASSVETKPGASKVATKTKATGASKKLKKATGASKKSVKTPKKAKKPAATRKSSKNPKKPKTVKPKKVAKSPAKAKAVKPKAAKARVTKPKTAKPKKAAPKKK
RPL6	Q02878	37.21	MAGEKVEKPDTKEKKPEAKKVDAGGKVKKGNLKAKKPKKGKPH
KRR1	Q13601	33.90	PPKEKPIVKPKEASTETKIDVASIKEKVKKAKNKKLGALTAEEIALKMEADEKKKKKK
MESD	Q14696	30.23	GKGGGSKEKNKTKQDKGKKKKEGDLKSRSSKEENRAGNKREDL
HSP90AA5P	Q58FG0	31.58	NKQVSDAEAEKKEDKRKKKKESNDKPEIEDVGSDEEEEKKDADKKKKKSKEKYIDQE
ARSJ	Q5FYB0	33.33	PSKNQAEKKQKKSKKKKKKQQKAVSGSTCHSGVTCG
H2BC19P	Q6DRA6	35.48	MPEPAKFAPAPKKGSKKAVTKAQKKDGKKRK
IQCA1	Q86XH1	35.29	ELKNLKLAVDRERERPVKAGKKKDKKGKKGKKKEKKAKKDKDLTADRTIES
FAM133A	Q8N9E0	30.30	NESSSKKRERKKKRKKKSCRSSSSSSSSDSSSSSSDSEDEEKKQGKRRKKKKNRSYKSSQSSTHESESESKESVKKKKKSKDETEKEKDVRSLSKKRKKSYP<br>DDKPLSSESSSESDYEEDVQAKKKRRCEEREQAKEKVKKKKKKQHKKHSKKKKKKSGSSHKSR
BBOF1	Q8ND07	33.33	MPSKGKDKKKGKSKGKDTKKLIKTDESVVDRAKANA
RP9	Q8TA86	47.83	SSSSEGKEKHKKKKKKEKHKKRKKEKKKKKKRKHKSSKSNEGSDSE
STT3B	Q8TCJ2	31.91	TLDHKPRVTNIFPKQKYLSKKTTKRKRGYIKNKLVFKKGKKISKKTV
CCDC7	Q96M83	33.96	LQKLKDKEKTKPTNNRTKKAVKTVKKKDKGKSEDSEKKMSPEKEFKIKEDLDQ
SEC62	Q99442	40.26	VMKMKYDKDIKKEKDKGKAESGKEEDKKSKKENIKDEKTKKEKEKKKDGEKEESKKEETPGTPKKKETKKKFKLEPH
PSMD1	Q99460	30.61	VLSITAKAKKKEKEKEKKEEEKMEVDEAEKKEEKEKKKEPEPNFQLLDN
RBM28	Q9NW13	41.67	AKKKLRNKTKEKGKNENSECPKKEPKAKKAKVADKK
TMA7	Q9Y2S6	31.25	MSGREGGKKKPLKQPKKQAKEMDEEDKAFKQKQKEEQKKLEELKAKAAGKGPLATGGIKKSGKK

B

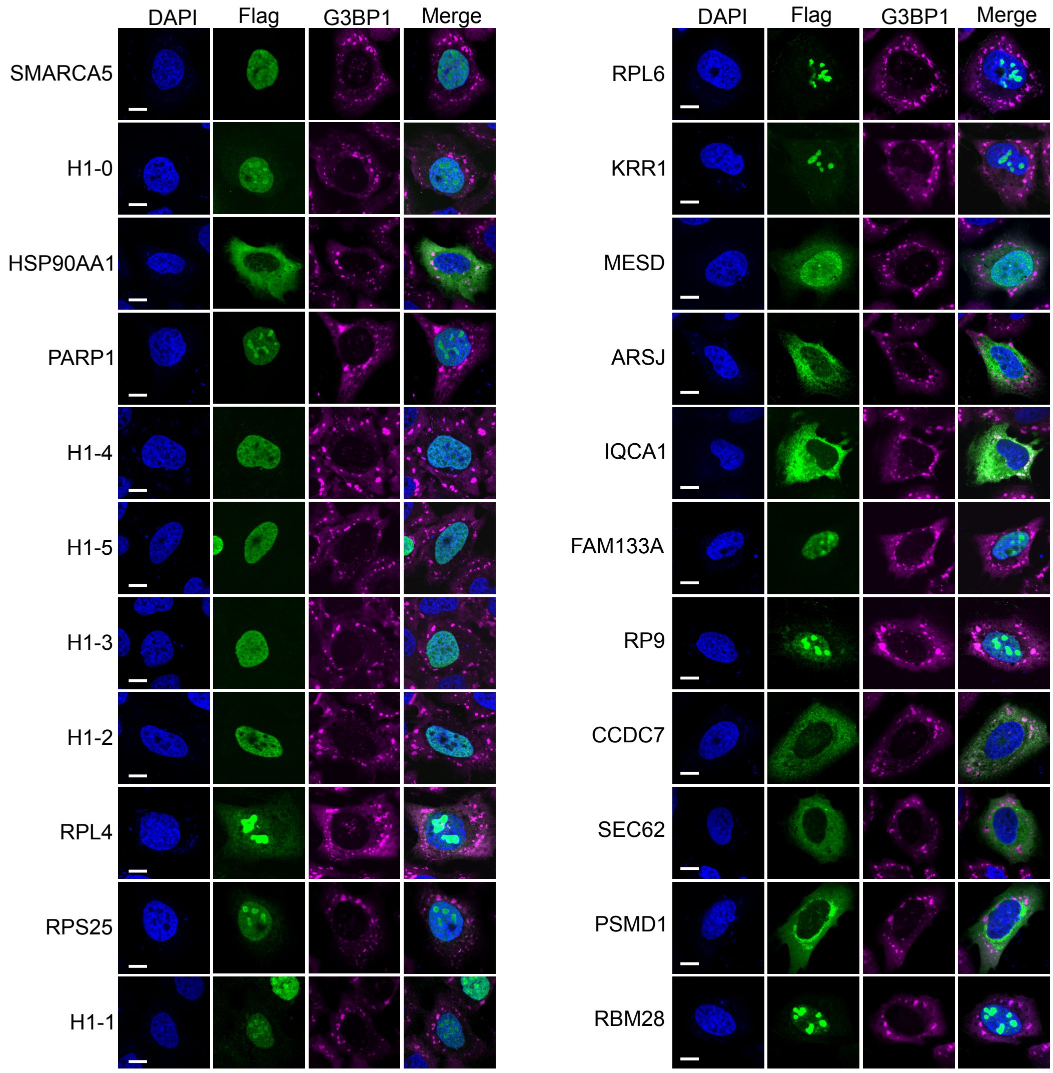

Supplement: Supplementary file 9 — Supporting File 9: advs74010‐sup‐0009‐FigureS8.pdf. [file ADVS-13-e17009-s008.pdf]
